# Genetic causes of the lissencephaly spectrum: insights from chromosomal microarray and clinical/whole-exome sequencing

**DOI:** 10.3325/cmj.2026.67.144

**Published:** 2026-06

**Authors:** Ana-Maria Meašić, Katarina Vulin, Adriana Bobinec, Leona Morožin Pohovski, Ivona Sansović, Morana Mikloš, Mijana Kero, Ana Tripalo Batoš, Ljubica Odak, Ingeborg Barišić

**Affiliations:** 1 Department of Medical and Laboratory Genetics, Endocrinology and Diabetology with Daily Care Unit, Children’s Hospital Zagreb, University of Zagreb, School of Medicine, Zagreb, Croatia; 2 University of Applied Health Sciences, Zagreb, Croatia; 3 Department of Pediatrics, University Hospital Center Split, Split, Croatia; 4 Department of Department of Paediatric Radiology, Children's Hospital Zagreb, Zagreb, Croatia; 5 Polyclinic Sinteza, Zagreb, Croatia

## Abstract

**Aim:**

To determine the diagnostic yield of comprehensive genetic testing in patients with neuroimaging findings suggestive of lissencephaly spectrum disorders and to characterize novel pathogenic variants contributing to the genetic architecture of the spectrum.

**Methods:**

We reviewed clinical and genetic findings of 23 patients with neuroimaging features suspected of the lissencephaly spectrum who underwent genetic testing at the Children’s Hospital Zagreb between 2016 and 2025. Clinical data were obtained from medical records and outpatient assessments by clinical geneticists. Genetic testing included chromosomal microarray, clinical exome sequencing, and whole-exome sequencing.

**Results:**

A molecular diagnosis was established in 15 of 23 patients (65.2%). The pathogenic variants involved genes related to microtubule function (*DCX*, *TUBA1A*, *TUBB2B*, *DYNC1H1*) and variants in transcriptional and regulatory genes (*FOXG1*, *WDR62*). Four novel (likely) pathogenic variants were detected in well-established lissencephaly genes (*DCX*, *TUBA1A*, *FOXG1,* and *WDR62*). Although most cases involved single-gene variants, three patients had pathogenic copy number variants (1q43q44, 22q11.21, and Xq22.3q23 deletions).

**Conclusion:**

Exome sequencing when used as a first-line test, complemented by chromosomal microarray, provides a high diagnostic yield in patients with lissencephaly spectrum disorders. This integrated approach facilitates precise diagnosis, informs prognosis, enables targeted follow-up, and supports comprehensive genetic counselling.

Lissencephaly is a rare malformation of cortical development (MCD) caused by abnormal neuronal migration. It represents a spectrum that includes agyria, pachygyria, and subcortical band heterotopia (SBH) (1). Neonatal presentation is generally unremarkable, but affected individuals later exhibit hypotonia, poor feeding, delayed motor milestones, and drug-resistant epilepsy (2). This leads to severe developmental delay, profound intellectual disability, and spastic quadriparesis (3).

On brain magnetic resonance imaging (MRI), lissencephaly is characterized by a thickened cerebral cortex and reduced gyration (4). In agyria, its most severe form, the brain appears smooth. More commonly, some shallow sulci are present, resulting in pachygyria, with broad, coarse gyri (5). SBH is a band of heterotopic gray matter beneath the cortex, separated from the cortex and the ventricles by normal-appearing white matter (6). Lissencephaly can co-occur with severe microcephaly (MIC) and/or additional brain malformations. According to the imaging-based classification proposed in 2017, there are 21 distinct patterns of lissencephaly. Identifying different patterns and associated brain malformations provides guidance in predicting the probable causative gene (4).

Lissencephaly is a predominantly genetic disorder, with at least 31 genes currently known to be associated with the condition (7,8). It was first linked to a genetic abnormality in 1983, when a 17p13.3 deletion was identified as the underlying cause of Miller-Dieker syndrome (9). The first gene identified in association with lissencephaly was *PAFAH1B1* (also known as *LIS1*), which remains the most frequently involved gene in affected individuals, followed by *DCX* (7,10,11). Pathogenic variants in *TUBA1A* and *DYNC1H1* are also relatively frequent (7,12,13).

Despite increasing knowledge of the genetic underpinnings of lissencephaly, the full spectrum of causative variants remains incompletely defined. In this study, we applied a diagnostic strategy comprising chromosomal microarray (CMA), clinical exome (CES), and whole-exome sequencing (WES) to 23 individuals with neuroradiologically confirmed lissencephaly spectrum (LIS). The aim of the study was to determine the diagnostic yield of comprehensive genetic testing in patients with LIS disorders and to characterize eventual novel pathogenic variants contributing to the genetic architecture of these conditions.

## Patients and methods

### Patients

We reviewed the medical records of 23 patients with LIS disorders who underwent genetic testing at the Children's Hospital Zagreb between 2016 and 2025. Inclusion criteria involved brain MRI findings consistent with LIS (agyria, pachygyria, and SBH) or a simplified gyral pattern representing an atypical LIS phenotype. Clinical data were obtained from medical records and examinations performed at the clinical genetics outpatient clinic. The study was approved by the Ethics Committee of the Children's Hospital Zagreb (02-23/13-2-18). Informed consent was obtained from all patients or their parents/guardians.

### Methods

**Figure 1:**
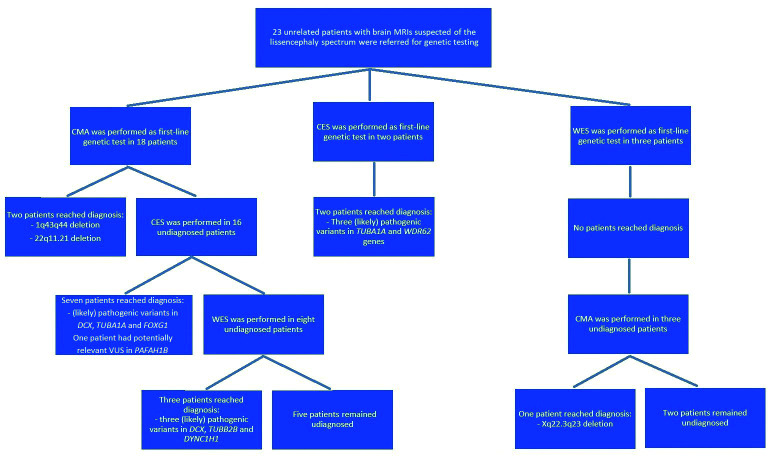
Diagnostic workflow and testing results of patients with brain MRIs suspected of lissencephaly spectrum. *MRI – magnetic resonance imaging; CMA – chromosomal microarray; CES – clinical exome sequencing; WES – whole exome sequencing. The figure reflects the sequence of diagnostic results, not necessarily the order in which tests were ordered.

In this retrospective study, genetic testing included CMA, CES, or WES performed on genomic DNA extracted from peripheral blood. From 2016 to 2025, CMA and CES were often ordered concurrently. Due to limited sequencing capacity and known shorter turnaround time, CMA results were typically available first (14,15). CMA also enabled rapid exclusion of the recurrent 17p13.3 deletion, a common cause of lissencephaly. In unresolved cases, CMA was generally followed by CES or WES (Figure 1). Parental segregation analysis used the same method as the proband analysis (CES for CES probands, trio WES for WES probands), except for patient P12, where targeted Sanger sequencing was required due to Next-Generation Sequencing mapping limitations.

CMA was performed to detect copy number variants (CNVs) using SurePrint G3 Unrestricted CGH ISCA v2 or GenetiSure Cyto CGH 8x60K (Agilent Technologies, Santa Clara, CA, USA). Data were analyzed using Agilent CytoGenomics software (GRCh37). CNV reporting thresholds were as follows: ≥3 consecutive probes, ≥50 kb for deletions, ≥100 kb for duplications, with log2 ratios≤-0.25 (deletions) and≥+0.25 (duplications).

CES and WES were used to identify single-nucleotide variants and small insertions and deletions (≤50 bp) within coding exons and ±20 bp from exon-intron boundaries. CES was performed using the TruSight One Sequencing Panel (GRCh37), and WES using the Exome v1.2 (CEX) (GRCh37) and Exome 2.5 (GRCh38) panels (Illumina, San Diego, CA, USA). CES was bioinformatically analyzed using three different workflows: Illumina Local Run Manager DNA Enrichment (BWA-MEM/GATK), DRAGEN Enrichment, and the Genomize-SEQ pipeline (BWA/FreeBayes). The Genomize-SEQ pipeline was also used for WES. Mean coverage was ≥100 × for CES and Exome 2.5, and ≥50 × for CEX, with ≥95% of target bases covered at ≥20 × . Variants were filtered using Human Phenotype Ontology (HPO)-driven prioritization, excluding (likely) benign variants and those with MAF>1%.

Sanger sequencing of *TUBB2B* and *TUBB2A* exon 4 was performed in patient P12 and her parents using a shared forward primer (5′TGTTCTTGGAGTCGAACATCTG3′) and gene-specific reverse primers (*TUBB2B* – 5′TACTGCATTGACAACGAGGC3′; *TUBB2A* – 5′TACTCCATTGATAACGAGGC3′) (Metabion, Planegg-Martinsried, Germany). In our standard workflow, Sanger confirmation is not routinely performed for exome-detected variants meeting quality criteria (coverage ≥20 × , unambiguous mapping); 15 of 16 exome-detected variants are accepted without Sanger validation. Patient P12 was the only exception due to high sequence homology between *TUBB2B* and *TUBB2A*.

Variant interpretation followed the guidelines of the American College of Medical Genetics and Genomics and the Association for Molecular Pathology (ACMG/AMP), the Clinical Genome Resource, and the Association for Clinical Genomic Science (16-18). Public databases and resources (gnomAD, https://gnomad.broadinstitute.org/; ClinVar, https://www.ncbi.nlm.nih.gov/clinvar/) were used to assess population frequencies and prior variant reports (19,20). For *in silico* splice prediction SpliceAI, Pangolin (https://spliceailookup.broadinstitute.org/) and dbscSNV (https://sites.google.com/site/jpopgen/dbNSFP) were used (21-23). All databases and tools were accessed in January 2026. Only (likely) pathogenic variants according to ACMG/AMP guidelines were considered clinically relevant.

## Results

### Cohort overview

Patients’ clinical presentation, including brain MRI findings, is summarized in Table 1. Of the 23 patients, 21 underwent CMA, 18 CES, and 11 WES (Figure 1). MCDs other than LIS were identified in eight patients, most commonly polymicrogyria (PMG) (n = 6). MIC was observed in 11 patients, and 13 patients had additional central nervous system malformations. All patients had neurodevelopmental delay (one patient was too young for assessment) ranging from mild to severe. Epilepsy was confirmed in 14 patients, and cerebral palsy was diagnosed in eight patients.

**Table 1:** Clinical findings in patients with the lissencephaly spectrum*

Patient ID	Sex	Age (years)	MCD	Other CNS anomalies	Additional findings	Neurodevelopmetal delay	Epilepsy	Cerebral palsy
P1	F	0.5	Thickened frontal cortex with simplified gyral pattern; MIC	Delayed cerebral and cerebellar myelination; CC agenesis; Reduced volume of frontal lobes; Cavum vergae and cavum septi pellucidi	ASD; Mild pulmonary valve stenosis	Mild	No	No
P2	M	4	Pachygyria; PMG	Ventriculomegaly	ASD II; Asymmetric IVS hypertrophy; Amblyopia; Hypermetropia; Astigmatism	Severe	Yes	No
P3	F	18.5	SBH	No	Microhematuria; Iron-deficiency anemia; Left-sided hearing loss	Moderate	Yes	No
P4	F	2.5	SBH	No	Hypermetropic astigmatism	Moderate	Yes	No
P5	F	10.5	SBH	Optic nerve atrophy; Intracranial hemorrhage	Esotropia; Overactive bladder	Moderate	No	No
P6	M	9	Pachygyria (bilateral, predominantly frontal); SBH	No	No	Mild	No	No
P7	F	3.5	SBH; MIC	Ventriculomegaly with parenchymal atrophy; Perinatal hemorrhage	Right esotropia	Mild	Yes	No
P8	M	4	LIS	No	Exotropia; Astigmatism; Hypermetropia	Severe	Yes	Yes
P9	F	16.5	Simplified gyral pattern; MIC	CC agenesis; Ventriculomegaly; Dandy Walker	No	Severe	Yes	Yes
P10	M	17.5	LIS; Pachygyria	No	Chronic respiratory insufficiency	Severe	Yes	Yes
P11	F	7.5	Simplified gyral pattern; MIC	CC agenesis; Dandy Walker	No	Moderate	Yes	Yes
P12	F	7	Pachygyria (bilateral); MIC	Vermian hypoplasia	Hypermetropia; Strabismus	Mild	No	Yes
P13	M	14.5	LIS; Pachygyria; PMG; MIC	CC hypoplasia	Hypermetropia; Astigmatism	Moderate	Yes	No
P14	M	15.5	Pachygyria (frontal bilateral); MIC	Absent anterior commissure; Diffuse white matter reduction with secondary ventriculomegaly	Astigmatism; Ambliopia; Cryptorchidism	Severe	Yes	Yes
P15	M	12.5	Pachygyria (frontal bilateral, right temporal/Sylvian); MIC	Hypoplasia of the frontal lobe; Enlarged inner CSF spaces	Esotropia; Bilateral cryptorchidism	Severe	Yes	Yes
P16	F	17	Pachygyria; PVNH; MIC	No	No	Severe	Yes	No
P17	M	12.5	Simplified gyral pattern (bilateral, frotanl and temporal); PMG (bilateral temporal; MIC	No	Cryptorchidism	Severe	Yes	Yes
P18	M	2.5	Pachygyria (parietal, 7-8 mm)	Mild right cerebral hypoplasia (symmetrical morphology); Right monoventriculomegaly; Absent septum pellucidum	Bilateral esotropia; Cryptorchidism	Mild	No	No
P19	M	7	Pachygyria; PMG	No	Hearing impairment	Mild	No	No
P20	M	0.5	PMG (bilateral, predominantly temporal and parietal); SBH (suspected, bilateral)	White matter reduction (temporal and parietal); Occipital horn ventriculomegaly; Widened subarachnoid spaces	Chronic respiratory insufficiency; Laryngomalacia; Patent foramen ovale	NA	No	No
P21	F	8	Pachygyria/PMG (right hemisphere)	No	Hearing impairment; VUR grade II	Mild	No	No
P22	M	6	SBH (bilateral frontoparietal and right insular); FCD (suspected)	No	No	Mild	Yes	No
P23	M	4.5	LIS; Pachygyria; MIC	Delayed myelination	Esotropia; Hydrocele/Left inguinal hernia	Mild	No	No

*Abbreviations: ID – patient identifier; F – female; M – male; MCD – malformations of cortical development; MIC – microcephaly; PMG – polymicrogyria; SBH – subcortical band heterotopia; LIS – lissencephaly; PVNH – periventricular nodular heterotopia; FCD – focal cortical dysplasia; CNS – central nervous system; CC – corpus callosum; CSF – cerebrospinal fluid; ASD – atrial septal defect; IVS – interventricular septum; VUR – vesicoureteral reflux; NA – not available; NDD – neurodevelopmetal delay.

### Genetic findings

**Figure 2:**
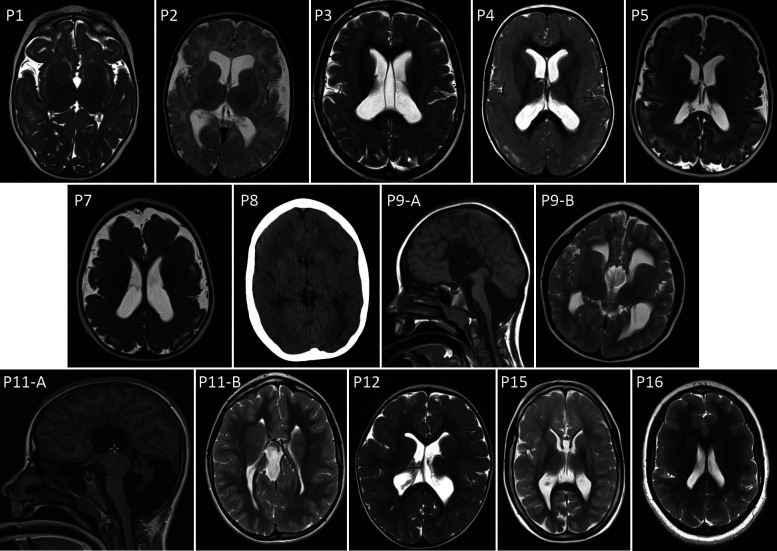
Brain MRIs of 12/15 diagnosed cases and a CT scan of patient with an identified VUS variant showing lissencephaly spectrum disorders: sagittal T1-weighted MRIs of patients P9-A and P11-B; axial T2-weighted MRIs of patients P1-P5, P7, P9-B, P11-B, P12, P15, P16; a CT scan of patient P8. *Abbreviations: MRI – magnetic resonance imaging; CT – computed tomography; VUS – variant of uncertain significance.

A genetic diagnosis was established in 15/23 patients (65.2%), with a median age of 10.5 years at the time of diagnosis (range: 0.5-18.5 years). Eight patients (34.8%) remained undiagnosed, although one had a potentially relevant variant of uncertain significance (VUS). Diagnostic yields were as follows: CMA 3/21 (14.3%), CES 9/18 (50%), and WES 3/11 (27.3%). These numbers are descriptive only due to sequential testing (Figure 1). Brain MRI images of 12/15 diagnosed patients and a computed tomography scan of one patient with potentially relevant VUS are presented in Figure 2.

Three heterozygous pathogenic CNVs were identified (Table 2). Patient P1 had a *de novo* 1q43q44 deletion, P2 harbored a 22q11.21 deletion, and P3 had an Xq22.3q23 deletion.

**Table 2:** Pathogenic CNVs detected with chromosomal microarray*

Patient ID	CNV	Genomic coordinates (GRCh37)	Size (Mb)	Inheritance
P1	1q43q44 del	240871275-249212668	8.35	*de novo*
P2	22q11.21 del	18894835-21440514	2.54	unknown
P3	Xq22.3q23 del	107136060-110810427	3.67	unknown

*Abbreviations: ID – identification; CNV – copy number variant; del – deletion; GRCh37 – Genome Reference Consortium Human Build 37; Mb – mega bases.

Thirteen different (likely) pathogenic variants were identified across seven genes in 12 patients and one VUS in one patient (Table 3). Four patients harbored *DCX* variants, including heterozygous nonsense, frameshift, and missense variants, as well as one hemizygous missense variant. One heterozygous splice-site variant was observed in *PAFAH1B1*. Heterozygous missense variants in *TUBA1A* were identified in three patients. Single heterozygous missense variants were observed in *TUBB2B* and *DYNC1H1*. Two patients had a heterozygous *FOXG1* variant, including a frameshift and a nonsense variant. Finally, one patient harbored two heterozygous frameshift variants in *WDR62*.

**Table 3:** Variants detected with clinical and whole exome sequencing*^†^

Patient ID	Gene	Transcript	c.DNA change	Protein change	Zygosity	Inheritance	ACMG classification
P4	*DCX*	NM_000555.3	c.508C>T	p.(Arg170Ter)	Het	unknown	P
P5			**c.567_577del**	**p.(Asp189GlufsTer7)**	Het	*de novo*	P
P6			c.775C>T	p.(Arg259Cys)	Hem	unknown	LP
P7			c.971T>C	p.(Phe324Ser)	Het	unknown	LP
P8	*PAFAH1B1*	NM_000430.3	**c.117 + 3A>G**	**p.?**	Het	unknown	VUS
P9	*TUBA1A*	NM_006009.4	c.1007A>G	p.(Lys336Arg)	Het	unknown	LP
P10			c.1204C>T	p.(Arg402Cys)	Het	unknown	P
P11			**c.1262C>T**	**p.(Ala421Val)**	Het	non-maternal	LP
P12	*TUBB2B*	NM_178012.5	c.743C>T	p.(Ala248Val)	Het	*de novo*	P
P13	*DYNC1H1*	NM_001376.5	c.10030C>T	p.(Arg3344Trp)	Het	unknown	P
P14	*FOXG1*	NM_005249.5	c.460dup	p.(Glu154GlyfsTer301)	Het	*de novo*	P
P15			**c.625G>T**	**p.(Glu209Ter)**	Het	unknown	LP
P16	*WDR62*	NM_001083961.1	**c.1207del** c.2864_2867del	**p.(His403ThrfsTer27)** p.(Asp955AlafsTer112)	Compound het	inherited (one maternal, one paternal)	LP P

*Novel variants are indicated in bold.

†Abbreviations: ID – identification; Het – heterozygous; Hem – hemizygous; ACMG – American College of Medical Genetics and Genomics; P – pathogenic; LP – likely pathogenic; VUS – variant of uncertain significance.

Parental testing was available for a subset of patients and included parental CES for CES probands and trio WES for WES probands. Inheritance data are summarized in Tables 2 and 3. *De novo* occurrence was confirmed for the *DCX* variant p.Asp189GlufsTer7, the *FOXG1* variant p.Glu154GlyfsTer301, and the *TUBB2B* variant p.Ala248Val (the latter by parental Sanger sequencing). The *WDR62* variants p.His403ThrfsTer27 and p.Asp955AlafsTer112 were confirmed *in trans* by parental CES. For the patient harboring the *TUBA1A* variant p.Ala421Val, the mother, who was the only family member available for testing, tested negative. Variants without parental testing are reported as having unknown inheritance.

Given the high sequence homology (96%) between *TUBB2B* and *TUBB2A* in exon 4, gene-specific Sanger sequencing in patient P12 and her parents confirmed the *TUBB2B* variant c.743C>T (p.Ala248Val) and its *de novo* occurrence.

## Discussion

In this study, we investigated genetic causes of lissencephaly in patients with clinical and radiological suspicion of neuronal migration disorders. This study reflects real-world clinical practice, during which limited sequencing capacity resulted in a non-uniform testing approach, making direct comparison of diagnostic yields between CMA, CES, and WES methodologically inappropriate. A genetic diagnosis was achieved in 65.2% of cases. This yield aligns with recent reports employing comprehensive genomic sequencing in MCD, though lower than the 75%-80% reported in larger cohorts (7,24). This difference may be explained by several factors, including patient selection, cohort size, and technical limitations in detecting non-coding variants, balanced rearrangements, and low-level mosaicism.

CMA was mostly the first-line genetic test applied in our cohort. While CNVs represent a less frequent cause of lissencephaly compared with single-gene variants, their contribution is well documented (7,25). Three pathogenic CNVs detected in our cohort are discussed below with respect to their contribution to the development of LIS.

Patient P1 carried a 1q43q44 deletion and presented with MIC, corpus callosum agenesis, and neurodevelopmental delay consistent with 1q43q44 microdeletion syndrome, although epilepsy had not yet manifested by one year of age. The phenotype is primarily caused by deletion of *AKT3*, *HNRNPU*, and *ZBTB18* (26). MRI showed a simplified gyral pattern with cortical thickening. Only one case of 1q43q44 microdeletion associated with a neuronal migration disorder (pachygyria) has been reported so far (27). *HNRNPU* disruption affects gene expression and splicing of numerous downstream targets involved in cell viability and motility, contributing to neural progenitor cell loss and impaired neuronal migration (28). Loss-of-function (LoF) of *ZBTB18* impairs radial neuronal migration and cortical lamination in murine models (29). Deletion of these genes may play a role in neuronal migration disorders, although a direct causal link between this CNV and LIS remains to be established.

Patient P2 harbored a recurrent 22q11.21 deletion associated with DiGeorge syndrome. This syndrome is characterized by a wide phenotypic spectrum (including cardiac, endocrine, and craniofacial abnormalities), as well as brain malformations, neurodevelopmental delay, epilepsy, and facial dysmorphia observed in P2 (30). PMG and cortical dysplasia are commonly reported in patients with 22q11.21 deletion, whereas pachygyria, especially combined with PMG, is rare, and its association with 22q11.21 remains controversial (31,32). *TBX1* haploinsufficiency affects cortical development in murine models (33). Thus, while an association between 22q11.21 deletion and PMG is well established, its link to pachygyria requires further validation.

Deletion of Xq22.3q23, detected in P3, is classified as an X-linked contiguous gene deletion syndrome. Affected male patients exhibit microhematuria, renal failure, hearing loss, ocular changes, intellectual disability, midface hypoplasia, and elliptocytosis, whereas female carriers usually show only mild clinical features, such as microscopic hematuria. The disorder is associated with deletions of *COL4A5*, *AMMECR1*, and *ACSL4* (34). Crucially, this deletion also encompasses *DCX,* the second most common cause of lissencephaly and the primary cause of X-linked subcortical band heterotopia. Only one female patient with an Xq22.3q23 deletion involving *DCX* and SBH similar to P3 has been reported so far (35). The SBH phenotype in P3 is likely attributable to *DCX* haploinsufficiency, while the contiguous deletion accounts for additional features such as microhematuria and hearing loss.

The majority of findings in our cohort were detected by exome sequencing (CES/WES), which shows both the predominance of single-gene defects in the genetic etiology of lissencephaly and higher diagnostic yield of exome sequencing, as reported in other neurodevelopmental disorders (36). Identified variants mainly affected genes involved in microtubule organization and functioning, including genes for microtubule-associated proteins (*DCX* and *PAFAH1B1*), tubulin genes (*TUBA1A* and *TUBB2B*), and a gene for microtubule motor protein (*DYNC1H1*). These results confirm the importance of microtubule stability and organization in neuronal migration during cortical development (37).

In our cohort, the most frequently affected gene was *DCX*, which accounted for the majority of single-gene pathogenic variants (4/15 diagnosed cases). Among four patients with *DCX* variants, three female patients presented with SBH. The male patient exhibited predominantly anterior pachygyria with SBH, consistent with the more severe brain malformations typically observed in hemizygous males. This aligns with the fact that *DCX* is the second most common cause of lissencephaly after *PAFAH1B1*, and the main cause of X-linked classic lissencephaly in men and SBH in women (7,38,39). Three of four *DCX* variants were previously reported, which supports their pathogenicity (38,40-42). The frameshift variant p.Asp189GlufsTer7 in P5 is a new truncating mutation in a gene where LoF is a mechanism of pathogenicity, suggesting a negative effect.

The phenotype of female patients with *DCX* variants was consistent with typical clinical presentation in women. The male patient (P6) carrying the p.Arg259Cys showed a mild clinical phenotype. This may be because missense *DCX* variants are associated with a milder clinical presentation than LoF variants (39). While LoF mutations occur randomly throughout the DCX protein, pathogenic missense variants cluster within conserved tandem N-terminal and C-terminal doublecortin (DC) domains (38). Missense variants within the C-DC domain may impact function less severely than variants affecting the N-DC domain (43). Furthermore, p.Arg259Cys variant resides on the surface of the C-DC domain, and surface missense variants typically cause a milder phenotype than buried ones, which are predicted to impair protein stability (38,44).

A non-canonical splice-site variant, c.117 + 3A>G in *PAFAH1B1*, was identified in P8. This variant is novel, not previously reported in association with LIS, and absent from gnomAD. *In silico* splicing tools predict donor-site disruption, particularly those optimized for non-canonical splice-site variants. While the evidence to classify c.117 + 3A>G as (likely) pathogenic is currently insufficient, splice-site variants are a known mechanism of pathogenicity in *PAFAH1B1* (45). Functional validation through RNA studies or minigene assays is necessary to confirm splicing disruption. The neuroimaging of P8 is consistent with a *PAFAH1B1-*associated phenotype supporting potential pathogenic relevance.

Pathogenic variants affecting tubulin genes were found in four patients. Tubulin genes are associated with tubulinopathies, a clinically heterogeneous group of disorders characterized by a broad spectrum of MCDs, including agyria-pachygyria, PMG, cortical gyral simplification, and MIC (4,46).

Patients P9, P10, and P11 harbored heterozygous missense variants in the C-terminal region of *TUBA1A*, a known hotspot region. Variants in P9 and P10 are known variants: p.Lys336Arg is reported in ClinVar, and p.Arg402Cys is a recurrent variant, because Arg402 represents the most frequently affected amino acid residue in *TUBA1A* (25,46). The p.Ala421Val variant in P11 is a new, likely pathogenic change, absent from public databases and only noted in a supplementary data set without clinical interpretation (47). Another missense variant at the same codon (p.Ala421Thr) is classified as likely pathogenic in ClinVar, supporting the significance of this residue.

A missense variant, p.Ala248Val in *TUBB2B*, was identified in P12. Although population databases report an apparently high allele frequency, these data fail quality control filters and are likely artifacts due to segmental duplications and high sequence similarity among beta-tubulin genes and the pseudogene, which may explain the conflicting classifications in ClinVar (48). For this reason, Sanger sequencing with gene-specific primers was essential to unambiguously map the variant to *TUBB2B.* Pathogenic *de novo* missense variants in *TUBB2B* are enriched in exon 4, affecting highly conserved residues, such as Ala248, which shows the functional importance of this region (49,50). The p.Ala248Val variant is a known pathogenic variant. Another missense variant affecting the same residue (p.Ala248Thr) is also classified as pathogenic, supporting the important role of Ala248 in *TUBB2B* function (50,51).

A heterozygous missense variant in *DYNC1H1* identified in P13 was already observed in individuals with features of *DYNC1H1*-related lissencephaly (7). *DYNC1H1* encodes the heavy chain of cytoplasmic dynein 1, a microtubule motor protein complex consisting of the N-terminal stem and C-terminal motor domains. The variant in P13 affects the motor domain associated with MCDs (52).

Besides genes affecting microtubules, we also identified (likely) pathogenic variants in *FOXG1* and *WDR62,* genes that are not directly related to microtubule dynamics but are involved in transcriptional and regulatory pathways critical for neuronal proliferation, differentiation, and cortical organization.

The LoF variants in *FOXG1* were identified in two patients: a recurrent frameshift variant in P14 and a novel nonsense variant p.Glu209Ter in P15. Both variants are truncating and located in the N-terminal region, predicted to disrupt the forkhead DNA-binding domain (53,54). *FOXG1* encodes a transcriptional repressor essential for early telencephalic development. Its disruption causes *FOXG1* syndrome, a severe neurodevelopmental delay characterized by congenital or postnatal MIC, movement disorders, hypotonia, neurobehavioral/psychiatric manifestations, and epilepsy (54). Neuroimaging findings show LIS disorders ranging from frontal pachygyria to a simplified gyral pattern, as seen in P14 and P15 (53). This pattern likely reflects the very early role of *FOXG1* in telencephalic patterning and cortical organization rather than a pure defect of neuronal migration.

Finally, in P16, two frameshift variants in *WDR62* were detected *in trans*, a novel variant p.His403ThrfsTer27 and a known variant p.Asp955AlafsTer112 (55). Recessive LoF pathogenic variants in *WDR62* are a well-established cause of MCDs, including MIC, pachygyria, schizencephaly, and PMG (56,57).

This study has several limitations, including a small cohort (n = 23) reflecting the rarity of LIS disorders. Seven patients remained without molecular diagnosis, despite comprehensive testing, which reflects the genetic heterogeneity of LIS disorders and the limitations of current diagnostic genetic tests. Low-level mosaicism, non-coding variants, and trinucleotide expansions, undetectable by CMA or CES/WES, could not be excluded. Functional validation of novel variants and the *PAFAH1B1* splice-site VUS was beyond the scope of the study.

In summary, a molecular diagnosis was established in approximately two-thirds of patients, with the majority of pathogenic findings affecting genes involved in microtubule organization and function, while the remaining 34.8% undiagnosed patients highlight diagnostic limitations of current testing options. The resolution of these cases will require the use of alternative tissues, targeted deep sequencing, whole-genome sequencing, RNA sequencing, or long-read technologies, together with international data sharing and collaborations (25,58).

Our findings further broaden the known mutational spectrum through the identification of four novel (likely) pathogenic variants. The inclusion of patients with simplified gyral patterns enabled the detection of causative variants beyond classic lissencephaly, supporting the concept of a broad spectrum. The predominance of single-gene variants detected by CES/WES supports the growing consensus that exome sequencing should be considered a first-tier test for LIS disorders. CMA remains valuable and should be retained in the diagnostic workflow, particularly for syndromic presentations. Future diagnostic algorithms may benefit from simultaneous CMA and WES to reduce diagnostic delays. This study illustrates the extensive genetic and phenotypic heterogeneity of LIS and emphasizes the importance of comprehensive genomic testing in patients with neuronal migration disorders, alongside careful neuroradiological and clinical phenotyping to guide interpretation.
